# Various forms of alcohol use and their predictors among pregnant women in post conflict northern Uganda: a cross sectional study

**DOI:** 10.1186/s13011-020-00337-8

**Published:** 2021-01-04

**Authors:** Apophia Agiresaasi, Goretti Nassanga, Gakenia Wamuyu Maina, Juliet Kiguli, Elizabeth Nabiwemba, Nazarius Mbona Tumwesigye

**Affiliations:** 1 College of Health Sciences, Makerere School of Public Health, Kampala, Uganda; 2 Department of Journalism and Communication, College of Humanities and Social Sciences, Kampala, Uganda

**Keywords:** Alcohol use, Pregnancy, Antenatal care, Post conflict, Uganda

## Abstract

**Background:**

Alcohol use during pregnancy has been associated with several births and developmental disorders. This study set out to determine the various forms of alcohol consumption among pregnant women and their predictors in post conflict Northern Uganda.

**Methods:**

In the months of May to June 2019, we conducted a cross sectional study among 420 pregnant women seeking antenatal care services at both Government and private health facilities in Gulu, Kitgum and Pader districts in Northern Uganda. We asked them about consumption of various alcoholic beverages. A three stage stratified cluster sampling approach was used and study participants randomly selected from health facilities of interest. We used descriptive statistics to estimate the prevalence of various forms of alcohol use. The chi- square test and logistic regression were used to assess associations of alcohol use among respondents and their socio - demographic and other characteristics.

**Results:**

Overall 99 women (23.6%) reported current alcohol use (any amount). Up to 11% (*N* = 11) of all drinkers were identified by the AUDIT to be women with problem drinking behavior, 8% (*N* = 8) of women reported hazardous drinking and only four (4%) were women with active alcohol dependent behavior. Predictors of maternal alcohol use included pre-pregnancy alcohol consumption, knowledge, attitude, education level, parity and residence.

**Conclusions:**

This study indicates that alcohol use (any mount) during pregnancy is high while alcohol dependence, problematic and hazardous drinking is low. Knowledge and attitude were important predictors of alcohol use. While alleviating alcohol use, development partners and relevant government departments should consider communication and other interventions that increase knowledge and risk perception on maternal drinking. Other risk factors that predict maternal drinking such as prior alcohol use, residence and parity should be mitigated or eliminated.

## Background

Uganda has the 7th highest rate of alcohol consumption in the Africa region according to the World Health organisation (WHO) (2018) report [1]. More so, some studies in Uganda have recorded higher prevalence of alcohol use in the general population in post conflict Northern Uganda compared to other regions in the country [2–4]. This has been attributed to armed banditry and traumatic stress resulting from the two- decade civil war between the Lord’s resistance Army and the Government of Uganda that displaced 90% of the region’s population [5]. It also disrupted the socio economic set up rendering many homeless and jobless [6]. After the war many women resorted to making alcohol as a source of livelihood given the demand for it but also to treat depression resulting from the war [7] Alcohol is also part and parcel of social gatherings in these communities and women, given their socially ascribed roles as caretakers are involved in serving and preparation hence at risk of using or abusing alcohol even during pregnancy [8]. Worse still, the vast majority (84%) of people in Northern Uganda are living below the poverty line [9] which is four times higher than the national average of 20%. The relationship between alcohol use, poverty and war has been well established. Because of these associations, we hypothesize that pregnant mothers in the lowest wealth quintile from conflict affected areas are more likely to drink than their other contemporaries. This has a bearing on the health of the mother and the baby.

Alcohol use during pregnancy has been linked to pre - term births (PTB), stillbirths, low birth weight (LBW) several birth defects and developmental disabilities known as Fetal Alcohol Spectrum Disorders (FASD) [10, 11].To-date, the World Health Organization recommends that pregnant women should completely abstain from alcohol as no amount of alcohol is considered safe for pregnancy [12]. Despite such recommendations Alcohol use among women in Africa is not uncommon.

In South Africa, prevalence of alcohol use (any amount) among pregnant women ranged from 3.2% in Johannesburg to 42.8% in Western Cape [13–15].. In Nigeria, prevalence varied from state to state. It ranged from 2.6% in Ogun state to 59.28% in South South Nigeria [16–19]. In Ghana, Bosomtwe district drinking (any amount) during pregnancy was reported at 20.4% [20] and 14.6% by another study [21]. In Eastern Africa, maternal alcohol use (any amount) varies. A recent study reported alcohol use during pregnancy at 15.1% in Dodoma Tanzania [22]. In Ethiopia maternal alcohol use was reported at 34% [23]. Whereas some studies have documented alcohol use during pregnancy in some parts of Africa, data for maternal alcohol use during pregnancy in post conflict settings remains notably scarce.

A systematic review and country specific studies have documented predictors of alcohol use during pregnancy across the world. Factors found to predict drinking during pregnancy include knowledge and attitude [24, 25] having disposable income [26], having a partner or friend who drinks [27] and Pre-pregnancy alcohol consumption [27–29]. Women with more children or higher number of previous pregnancies were more likely to drink during pregnancy than those with fewer children/pregnancies [30–32] .Being employed, having a higher income and higher social status have been reported to predict maternal drinking during pregnancy [31, 33]. Women with higher education were less likely to drink than women with less education [31].

In most maternal alcohol use related studies, predictors of alcohol use (any amount) have been examined. However predictors of frequent and or heavy alcohol use have not been assessed yet higher levels of alcohol use has been particularly associated with adverse effects on pregnancy. Without such data, it is difficult to adequately plan for any targeted alcohol exposed pregnancy interventions. To address this gap, this study investigated the prevalence of various forms of alcohol use during pregnancy and their predictors in post conflict northern Uganda.

## Methods

### Study area and design

From the month of May to June 2019, we conducted a cross sectional study of women attending the antenatal care at selected health facilities in Gulu, Kitgum and Pader districts in Post Conflict Northern Uganda. Gulu, Kitgum and Pader districts are served by 67, 28 and 19 public, private for profit and Private not for Profit health facilities providing Antenatal Care (ANC) services respectively. Agriculture is the main source of livelihood for locals in the three districts. Gulu district was the epi-centre of much of the fighting between the Ugandan army and the Lord’s Resistance Army (LRA). Kitgum and Pader districts also suffered many deaths and social disruption resulting from the two decade civil war that plagued the region.

We calculated the sample size using the Kish Lesley formulae. The degree of accuracy was set at 0.05, with estimated prevalence of alcohol consumption put at 30%. The minimum required sample size for the study was determined to be 322.6 but increased to 420 to cater for design effect.

This study used a multi stage cluster sampling method. Acholi sub-region was stratified into 8 regions consisting the 8 districts in the region. Simple random sampling was used to select the three districts of Gulu, Kitgum and Pader from the 8 districts in Acholi sub-region using paper lots. At the second stage, the health facilities providing antenatal care services in the three districts were randomly selected. For purposes of representativeness, all categories of health facilities in a given stratum were catered for. At the third stage, in each of the strata, the required number of respondents by health facility category were obtained randomly from the available facilities of the category of interest using systematic random sampling with the starting point obtained by simple ballot.

We evaluated Antenatal care attendees for eligibility and willingness to participate in the interview. To be eligible, women had to be at least 15 years old, at least be 8 weeks to 36 weeks gestation as estimated from the last normal menstrual period. Ability to speak any of the Acholi languages and or English. All those selected and willing to participate in the study were interviewed after having sought ANC services.

Data was collected using a computerized, Internet-based survey tool – Open Data Kit (ODK) and tablet computers fitted with checks. During the interview we asked women about alcohol consumption (any amount and any type). Frequency and quantity of alcohol consumed was also captured. This study assessed alcohol related problems using the World Health Organization’ Alcohol Use Disorders Identification Test (AUDIT) questionnaire which was administered as an oral interview. This tool is a 10-item self-report instrument that includes quantity and frequency of alcohol use and is designed to identify individuals currently experiencing alcohol problems [34]. Both the structured questionnaire and the AUDIT Questionnaire were piloted in neighboring Amuru district. The following measures were taken as one standard drink. i) a 285-ml bottle or can of beer, (ii) a 120-ml glass of wine (factory distilled or locally brewed), and [3] a 30-ml glass/tot of a spirit or gin (factory distilled or locally brewed). The questions in the AUDIT questionnaire were revised to reflect alcohol consumption by respondents since onset of pregnancy.

We interviewed women about their Socio demographic characteristics such as maternal age at first birth, level of education, marital status, occupation, age, level of income, residence, occupation, parity, religion, history of miscarriage, recreational drug use, smoking and other personal habits. We also asked women questions about their risk perception of using alcohol, knowledge of dangers of maternal drinking, past related behavior (pre-pregnancy alcohol use and sexual behaviors). Adequate knowledge was assumed if a woman mentioned 4 or more effects of alcohol, those who mention 2–3 effects were assumed to have fair knowledge and those who mention 0–1 were categorized as having poor knowledge.

### Data management and analysis

Data were cleaned and exported to Statistical Package for Social Sciences (SPSS) where coding and analysis was done. In the first stage of analysis, descriptive statistics were used to estimate the prevalence of various forms of alcohol use. The chi- square test was used to compare the drinking status of studied subjects with their socio - demographic and other characteristics. We did not do analyses for complex samples. For variables where expected count was less than 5 we reported the Fisher’s exact test *p*-value not the p-value from the chi-square test . Variables found to be significant at *P* ≤ 0.05 in the bivariate analyses were further modelled into the multiple logistic regression analysis.

Regarding the Audit, the hazardous alcohol use (questions 1 to 3) cut-off point was put at 6. The dependence (questions 4 to 6) cut-off was 4 and the harmful use (questions 7 to 10) cutoff was 7 points. Scores ≥8 were considered an indicator of problematic alcohol use [35].

Study participants were categorized as abstainers if they did not report alcohol consumption since on-set of pregnancy. Forms of alcohol consumption were 1) Light infrequent drinkers (those who reported alcohol use during current pregnancy but never took 4 drinks at any time), 2) Heavy infrequent drinkers (those who reported drinking 4 drinks less than monthly during current pregnancy), 3) Heavy moderately frequent drinking (reported drinking at least 4 or more drinks on an occasion at least monthly), 4) Heavy frequent drinking (reported drinking 4 drinks on an occasion at least weekly) and 5) Heavy regular drinking(reported drinking at least 4 drinks almost daily).

This study was approved by the Makerere University School of Public Health Institutional Review Board and the Uganda National Council for Science and Technology (UNCST) Ref SS 4938. Study participants were assured of the confidentiality of their responses and their written informed consent was sought before the interviews commenced. They were informed that they could choose not to answer any questions that they were not comfortable with or could withdraw from the interview at any time. In addition, the study sought clearance from the office of the District Health Officers (DHO) in each of the three districts of Gulu, Kitgum and Pader.

## Results

### Characteristics of study participants

Overall, 420 eligible women attending ANC in selected health facilities in Gulu, Kitgum and Pader districts in Northern Uganda participated in the study. Majority were aged 21–25 (56.2%). Up to 88.1% (*N* = 370) were either formally married of cohabiting. Sixty percent (*N* = 252) were Catholics. Slightly more than half (56.4%; *N* = 237) had attained primary level of education. Up to 57.9% (*N* = 243) were from rural areas. Most (*N* = 308; 73.3%) had a job from which they earned some income of whom (186; 44.3%) were farmers. This is presented in Table 1.

**Table 1:** Showing demographic characteristics of respondents and their drinking patterns

		ALCOHOL DRINKING								Chi-square/fishers test	*p*-value
		Never drank		Ever drank		Currently consuming Alcohol		Total			
Age Group	15–20	67	60.9%	22	20.0%	21	19.1%	110	100.0%		
	21–25	59	46.8%	43	34.1%	24	19.0%	126	100.0%	26.018^a^	.001
	26–30	39	37.1%	40	38.1%	26	24.8%	105	100.0%		
	31–35	22	38.6%	18	31.6%	17	29.8%	57	100.0%		
	36–45	6	27.3%	5	22.7%	11	50.0%	22	100.0%		
Highest Level of Education Attained	No formal education	18	41.9%	8	18.6%	17	39.5%	43	100.0%	17.996^a^	.006
	Primary	119	50.2%	70	29.5%	48	20.3%	237	100.0%		
	Secondary	47	46.5%	31	30.7%	23	22.8%	101	100.0%		
	Higher	9	23.1%	19	48.7%	11	28.2%	39	100.0%		
	Total	75	47.8%	61	38.9%	21	13.4%	420	100.0%		
Marital Status	Married	7	25.0%	3	10.7%	18	64.3%	157	100.0%		
	Single/separated/divorced	98	46.0%	56	26.3%	59	27.7%	28	100.0%	43.825	<.000
	Cohabiting	13	59.1%	8	36.4%	1	4.5%	213	100.0%		
	Other	109	43.3%	81	32.1%	62	24.6%	22	100.0%		
	Total	44	41.9%	33	31.4%	28	26.7%	420	100.0%		
Religion	Catholic	10	71.4%	2	14.3%	2	14.3%	252	100.0%		
	Protestant	30	61.2%	12	24.5%	7	14.3%	105	100.0%	**9.389**	**.146**
	Muslim	112	46.1%	65	26.7%	66	27.2%	14	100.0%		
	Pentecostal	71	45.5%	60	38.5%	25	16.0%	49	100.0%		
	Total	10	47.6%	3	14.3%	8	38.1%	420	100.0%		
Residence	Rural	84	45.2%	51	27.4%	51	27.4%	243	100.0%		
	urban	55	64.7%	20	23.5%	10	11.8%	147	100.0%	13.245	.009
	Small town/village	26	37.7%	26	37.7%	17	24.6%	21	100.0%		
	Big town/city	28	35.0%	31	38.8%	21	26.3%	9	100.0%		
Occupation	Farmer	84	43.5%	51	39.8%	51	51.5%	186	100.0%		
	Housewife	55	28.5%	20	15.6%	10	10.1%	85	100.0%	21.01	.002
	Retail business	26	13.5%	26	20.3%	17	17.2%	69	100.0%		
	Other	25	13.0%	21	16.4%	18	18.2%	64	100.0%		

^a^third of the respondents were aged 21–25 About a half had attained primary level education. Majority were either married or cohabiting Two thirds belonged to the Catholic religion

More than half (*N* = 225; 53.6%) were in second trimester of pregnancy and majority (*N* = 379; 90.2%) reported having one sexual partner in the last 1 year preceding the survey. Fewer women reported having ever had a premature birth (*N* = 30; 7.1%), an underweight baby (*N* = 25; 6.0%) or a still birth (*N* = 23; 5.5%). About a half (54.8%; *N* = 177) had at least two live children. Only three (0.7%) women had ever smoked or used tobacco while only two (0.5%) had ever used marijuana or other types of drugs. Poor knowledge (mentioned 0 to 1 effect of alcohol on mother and baby) was reported by (*N* = 234 (55.7%), fair knowledge (mentioned 2 to 3 effects of alcohol on mother and baby) by 41.9% and adequate knowledge (mentioned 4 or more effects of alcohol on mother and baby) by 2.4%. Slightly more than half the respondents had positive attitudes (Said alcohol was extremely important/very important in increasing health risks to mother and baby) (*N* = 228, 54.3%), few respondents had fair attitudes towards maternal alcohol use (said alcohol was moderately important in increasing health risks to mother and baby) (*N* = 58, 13.8%) and 134 respondents had Negative attitudes (said alcohol was not at all important /slightly important in increasing health risks to the mother and the baby (31.9%). Health workers were the most common source of information on drinking during pregnancy (*N* = 178, 75.1%) followed by friends (*N* = 65, 27.4%. This is presented in Table 2.

**Table 2:** Showing knowledge, attitudes and Gynaecologic characteristics of respondents and their drinking patterns

		ALCOHOL DRINKING								Chi-square	*p*-value
		Never drank		Ever drank		Currently consuming Alcohol		Total			
Live Children	One	36	42.9%	30	35.7%	18	21.4%	84	100.0%		
	Two	41	44.1%	37	39.8%	15	16.1%	93	100.0%	16.748^a^	.0328
	Three	38	45.2%	25	29.8%	21	25.0%	84	100.0%		
	Four	13	34.2%	11	28.9%	14	36.8%	38	100.0%		
	Five+	8	33.3%	4	16.7%	12	50.0%	24	100.0%		
Trimester	First trimester	56	50.9%	38	34.5%	16	14.5%	110	100.0%		
	Second trimester	100	44.4%	56	24.9%	69	30.7%	225	100.0%	16.666^a^	.0022
	Third trimester	37	43.5%	34	40.0%	14	16.5%	85	100.0%		
Sex Partners they have had in the last twelve months	One	181	47.8%	123	32.5%	75	19.8%	379	100.0%	31.3	< 0.000
	Two or more	12	29.3%	5	12.2%	24	58.5%	41	100.0%		
Knowledge of anything that could Harm baby during pregnancy	Yes	131	45.8%	100	35.0%	55	19.2%	286	100.0%	13.096	0.001
	No	62	46.3%	28	20.9%	44	32.8%	134	100.0%		
Attitude/perception of alcohol in increasing chances of health risks for wmn and babies	Negative attitude	49	36.6%	21	15.7%	64	47.8%	134	100.0%	68.498	< 0.000
	Fair attitude	25	43.1%	23	39.7%	10	17.2%	58	100.0%		
	Positive attitude	119	52.2%	84	36.8%	25	11.0%	228	100.0%		
Knowledge of the dangers of alcohol use during pregnancy	Poor knowledge	98	41.9%	59	25.2%	77	32.9%	234	100.0%	27.08	< 0.000
	Fair knowledge	89	50.6%	65	36.9%	22	12.5%	176	100.0%		
	Adequate	6	60.0%	4	40.0%	0	0.0%	10	100.0%		
My Family would not approve of me drinking while pregnant	Disagree	13	22.0%	4	6.8%	42	71.2%	59	100.0%	87.216	< 0.000
	Agree	180	49.9%	124	34.3%	57	15.8%	361	100.0%		
My Friends and Community wld approve of me drinking when pregnant	Disagree	178	48.4%	125	34.0%	65	17.7%	368	100.0%	59.689	< 0.000
	Agree	15	28.8%	3	5.8%	34	65.4%	52	100.0%		

^a^About a half of the respondents were in their second trimester of pregnancy, 22% had two live children. An overwhelming majority reported one sex partner in last twelve months preceding the survey. Most respondents agreed that their family and friends would not approve of them drinking during pregnancy

### Types of alcohol consumed, frequency and amount consumed

Of the 420 women surveyed, 194 (46.2%) reported lifetime alcohol abstinence. Slightly more than half (*N* = 226; 53.8%) reported that they have ever consumed alcohol, of these, 99 women (23.6%) reported alcohol use during current pregnancy and 10% (*N* = 33) reported that they stopped alcohol consumption after pregnancy recognition. The most common type of alcohol consumed was kwete (fermented maize and millet; ABV unknown) drunk by 50 women (50.5%) followed by beer drunk by 38 women (38.4%) (4–6% ABV). Twelve (12.1%) women drunk Arege (ABV unknown), seven drunk Lujulu (Fermented cassava, Sorghum and Maize; ABV unknown). Six consumed local waragi (ABV unknown). Two (2.0%) consumed dete (ABV unknown) and two (2.0%) consumed wine (12.5% ABV). Nine women (9.1%) consumed other alcoholic beverages. Up to 24.2% of alcohol consumers reported drinking two or more beverage types.

Among current drinkers,(*N* = 29; 29.3%) reported that they drink 3 to 4 drinks in a day. 69(69.1%) consumed 1 or 2 drinks in day. When asked how often they drunk 6 or more drinks on an occasion; (*N* = 14; 13.7%) said less than monthly, (*N* = 8; 7.1%) said monthly and (*N* = 2; 2%) said weekly.

A typology of drinkers was estimated [36] . Respondents were asked how often they consumed 4 or more drinks in a day; 33(33.3%) were light infrequent drinkers (reported alcohol use during current pregnancy but never took 4 drinks at any time), 29 participants (29.3%) reported heavy infrequent drinking (took 4 drinks less than monthly during current pregnancy), 24(24.2%), reported heavy moderately frequent drinking (drunk at least 4 or more drinks on an occasion at least monthly). Heavy frequent drinking(drinking 4 drinks on an occasion at least weekly) and heavy regular drinking(drinking at least 4 drinks almost daily) was less commonly reported by 11(11.1%) and (2%)respectively.

Up to 11% (*N* = 11) of all drinkers were identified by the AUDIT to be women with problematic alcohol use, 8%(*N* = 8) reported hazardous drinking and only four (4%) were women with alcohol dependence.

Almost a third of drinkers (29.3%) reported binge drinking less than monthly during pregnancy. Up to 24.2%, reported binging at least monthly. Weekly and daily binging was reported by 11(11.1%) and (2%) of drinkers respectively.

### Factors associated with alcohol consumption during pregnancy

Maternal alcohol consumption was associated with older age (*P* = 0.014), lack of formal education (*P* = 0.045) and pre-pregnancy alcohol consumption (*P* < 0.001). Knowledge was also associated with alcohol use during pregnancy. Women who reported that they have never received any information on drinking during pregnancy were more likely to drink during pregnancy (P < 0.001) as were women who said they had no knowledge of anything that could harm baby during pregnancy (*P* = 0.002). Gynecological factors associated with alcohol use during pregnancy include having a live birth (*P* = 0.004) and being in the second trimester (*P* = 0.001). Attitudes, prior and current behavior were strongly associated with alcohol use during pregnancy. Having two or more sex partners was associated with maternal alcohol use (*P* < 0.001). Also respondents who disagreed that their families would not approve of them drinking during pregnancy (*P* < 0.001) and those who agreed that their family and friends would approve of them drinking during pregnancy (*P* < 0.001) were more likely to drink than those who gave other responses. Women with negative attitudes (said alcohol was not all that important and those who said it was slightly important in increasing chances of health risks for women and babies were more likely to drink(*P* < 0.001). This is shown in Table 3.

**Table 3:** Showing factors associated with alcohol use during pregnancy

	Currently Drinking Alcohol				Chi-square	***p***-value
	No	%	Yes			
**Age**						
15–20	89	80.9%	21	19.1%		
21–25	102	81.0%	24	19.0%		
26–30	79	75.2%	26	24.8%	12.506	.014
31–35	40	70.2%	17	29.8%		
36–45	11	50.0%	11	50.0%		
**Highest Level of Education Attained**						
No formal education	26	60.5%	17	39.5%		
Primary	189	79.7%	48	20.3%	8.032	.045
Secondary	78	77.2%	23	22.8%		
Higher	28	71.8%	11	28.2%		
Ever Consumed Alcohol*						
No	193	99.5%	1	.5%	106.382	<.000
Yes	128	56.6%	98	43.4%		
Live Children*						
One	66	78.6%	18	21.4%		
Two	78	83.9%	15	16.1%	15.403	.004
Three	63	75.0%	21	25.0%		
Four	24	63.2%	14	36.8%		
Five+	12	50.0%	12	50.0%		
Trimester*						
First trimester	94	85.5%	16	14.5%		
Second trimester	156	69.3%	69	30.7%	13.641	.001
Third trimester	71	83.5%	14	16.5%		
**Religion**						
Catholic	190	75.4%	62	24.6%		
Protestant	77	73.3%	28	26.7%	3.723	.296
Muslim	12	85.7%	2	14.3%		
Pentecostal	42	85.7%	7	14.3%		
**Residence**						
Rural	177	72.8%	66	27.2%		
Urban	131	84.0%	25	16.0%	9.127	.010
Peri urban	13	61.9%	8	38.1%		
Sex Partners they have had in the last twelve months*						
One	304	80.2%	75	19.8%		
Two to Five	17	42.5%	23	57.5%	31.812	<.000
Six to ten	0	0.0%	1	100.0%		
Other	0	0.0%	0	0.0%		
Knowledge of anything that could Harm baby during pregnancy*						
Yes	231	80.8%	55	19.2%		
No	90	67.2%	44	32.8%	9.375	.002
Attitude/Perception of Alcohol use during pregnancy in increasing health risks to baby and mother*						
Negative attitude	70	52.2%	64	47.8%		
Fair attitude	48	82.8%	10	17.2%	64.927	<.000
Positive attitude	203	89.0%	25	11.0%		
My Family would not approve of me drinking while pregnant*						
Strongly disagree	10	76.9%	3	23.1%		
Disagree	7	15.2%	39	84.8%	111.843	<.000
Agree	216	81.5%	49	18.5%		
Strongly Agree	88	91.7%	8	8.3%		
My Friends and Community would approve of me drinking when pregnant*						
Strongly disagree	77	95.1%	4	4.9%		
Disagree	226	78.7%	61	21.3%		
Agree	15	31.3%	33	68.8%	70.853	<.000
Strongly Agree	3	75.0%	1	25.0%		
Ever received Information on drinking during pregnancy*						
Yes	228	88.0%	31	12.0%	50.486	<.000
No	93	57.8%	68	42.2%		

*p < 0.05 are variables significant at 5%

Significant bivariate variables included pre-pregnancy alcohol consumption, having live children knowledge, attitudes, number of sex partners and trimester of pregnancy

### Predictors of alcohol use during pregnancy

Among socio demographic factors examined, residence was predictive of maternal alcohol use. Women in urban areas were less likely to consume alcohol as compared to those in rural areas (OR 0.39; 95% CI: 0.17 to 0.91). Level of education was not predictive of alcohol use during pregnancy although women with secondary or higher level of education were more likely to drink (OR = 2.13; 95% CI: 0.42 to 3.21).

Also, women with 5 or more children were more likely to drink compared to those with one or no children (*P* = 0.018; OR = 5.4; 95% CI: 1.3 to 22.5). This variable was not significant for those with 2, 3, 4 children.

Knowledge and attitude were important predictors of maternal alcohol use in this study. Those who had never received any information about drinking during pregnancy are more likely to drink compared to those who have received the information (*P* = 0.002; OR = 4.08; 95% CI 1.6 to 10.1). Respondents who had no knowledge of anything that could harm the baby during pregnancy were less likely to drink (*P* = 0.004; OR = 0.24; 95% CI 0.09 to 0.64).

Women with positive or fair or positive attitude towards drinking during pregnancy (Said alcohol was moderately important/very important or extremely important in increasing chances of health risks for women and babies) were less likely to drink than those who said alcohol was not at all important in increasing chances of health risks for women and babies(*P* = 0.010; OR = 0.114; 95% CI = -0.02 to 0.59) Respondents who agreed to the question ‘my family would not approve of me drinking while pregnant’ were less likely to drink (*P* = 0.002; OR = 0.05; 95% CI 0.009 to 0.34). Number of sex partners was not a significant factor in predicting alcohol use during pregnancy although those with 2 or more partners have higher chances of drinking during pregnancy (OR = 2.6; 95% CI = 0.58 to 12.0). The remaining measures including, age, occupation and marital status all bear no significant association with self-reported alcohol use during pregnancy. These are shown in Table 4.

**Table 4:** Showing predictors of alcohol use during pregnancy

Variable	OR (95% CI)
**Level of Education**	
No formal education (Ref)	
Primary	0.9 (0.3 to 2.9)
Secondary	2.13 (0.51 to 8.8)
**Living Children**	
One (Ref)	
Two	1.2 (0.42 to 3.2)
Three	1.79 (0.66 to 4.8)
Four	3.3 (0.95 to 11.9)
Five and more	5.4 (1.3 to 22.5)^*^
**Residence**	
Rural (Ref)	
Urban	0.3 (0.17 to 0.91)
Peri urban	0.1 (0.019 to 0.84)
**Sex Partners in last Twelve Months**	
One (Ref)	
Two or more	1.9 (0.6 to 6.5)
**Knowledge of Anything that Could Harm Baby during Pregnancy**	
Yes (Ref)	
No	0.2 (0.09 to 0.64)^*^
**Attitude towards Alcohol Use During Pregnancy**	
Negative (Ref)	
Fair attitude	0.2 (0.05 to 0.65)
Positive attitude	0.16 (0.05 to 0.4)^*^
**My Family would not approve of me drinking while pregnant**	
Disagree (Ref)	
Agree	0.1 (0.04 to 0.36)^*^
**My Friends and Community would approve of me drinking when pregnant**	
Disagree (Ref)	
Agree	2.46 (0.84 to 7.1)
**Ever received Information on drinking during pregnancy**	
Yes (Ref)	
No	4.1 (1.64 to 10.1.)^*^
**Knowledge of Dangers of Alcohol Use during Pregnancy**	
Number of dangers	2.02 (0.7 to 5.6)
Number of Observations = 314	
LR chi2 (16) = 127.05	
Prob > chi2 = 0.0000	

^*^p < 0.05

Insignificant bivariate variables were not included in the model: Age, education and religion

### Predictors of frequent alcohol use and binge drinking

#### Frequent alcohol use

This study assessed predictors of frequent alcohol use (drinking at least 2 to 3 times a week). Mothers who agreed that ‘My Friends and Community would approve of me drinking when pregnant’ were more likely to drink 2 to 3 times a week as compared to those who had other responses (*P* = 0.017;OR = 7.95; 95% CI 1.45 to 43.3). Specific knowledge had a bearing on frequent alcohol use during pregnancy. Women were asked to list dangers of alcohol use to the mother and baby during pregnancy. The more dangers they mentioned the more they were likely to drink frequently (*P* = 0.008; OR = 11.2; 95% CI = 1.8 to 67.0). Other variables did not predict frequent alcohol use. This is presented in Table 5.

**Table 5:** Showing predictors of frequent drinking

Variable	OR(95% CI)
**Level of Education**	
No formal education (Ref)	
Primary	0.9 (0.09 to 9.5)
Secondary	1.3 (0.06 to 24.5)
Higher	0.8 (0.03 to 22.5)
**Living Children**	
One (Ref)	
Two	1.2 (0.19 to 7.6)
Three	2.3 (0.36 to 15.5)
Four	1.6 (0.20 to 13.0)
Five and more	0.36 (0.014 to 9.2)
**Residence**	
Rural (Ref)	
Urban	0.4 (0.08 to 2.27)
Peri urban	0.3 (0.01 to 6.5)
**Sex Partners in last Twelve Months**	
One (Ref)	
Two or more	2.6 (0.4 to 13.2)
**Knowledge of Anything that Could Harm Baby during Pregnancy**	
Yes (Ref)	
No	0.5 (0.1 to 2.6)
**Attitude towards Alcohol Use During Pregnancy**	
Negative (Ref)	
Fair attitude	1.3 (0.14 to 12.7)
Positive attitude	1.21 (0.14 to 10.5)
**My Family would not approve of me drinking while pregnant**	
Disagree (Ref)	
Agree	2.3 (0.47 to 11.3)
**My Friends and Community would approve of me drinking when pregnant**	
Disagree (Ref)	
Agree	7.9 (1.45 to 43.3)^*^
**Ever received Information on drinking during pregnancy**	
Yes (Ref)	
No	1.2 (0.22 to 6.74)
**Knowledge of Dangers of Alcohol Use during Pregnancy**	
No (Ref)	
Number of dangers	11.2 (1.8 to 67.02)^*^
Number of Observations = 80	
LR chi2 (17) = 32.41	
Prob > chi2 = 0.0134	
Pseudo R2 = 0.2956	

^*^p < 0.05

Significant variables for frequent drinking included knowledge and attitude

#### Binge/heavy drinking

Predictors of monthly, weekly and daily binge drinking were estimated. The effect of education on the likelihood of binge category was only significant with respect to women with secondary level and higher education (OR = 16.4; 95% CI:0.80 to 335.4). The odds ratios show that women with primary education were 6.2 times more likely to binge frequently. On the other hand, those with secondary and higher had 16.4 more odds of belonging to the binge category relative to those with no formal education. General knowledge was also predictive of binging. Those who said they had no knowledge of anything that could harm baby during pregnancy were more likely to binge frequently (*P* = 0.004; OR = 14.8; 95% CI 2.32 to 94.9). Women who agreed that ‘My Family would not approve of me drinking while pregnant were less likely to binge frequently than those who gave other responses (*P* = 0.04; OR = 0.2; 95% CI 0.04 to 0.93). This is shown in Table 6.

**Table 6:** Showing predictors of binge drinking

Variable	OR (95% CI)
**Level of Education**	
No formal education (Ref)	
Primary	6.2 (0.61 to 63.8)
Secondary	16.4 (0.80 to 335.4)
**Living Children**	
One (Ref)	
Two	0.13 (0.02 to 1.28)
Three	0.14 (0.02 to 1.38)
Four	0.09 (0.01 to 1.06)
Five and more	0.1 (0.01 to 2.32)
**Residence**	
Rural (Ref)	
Urban	1.5 (0.32 to 11.9)
Peri urban	0.03 (0.00 to 4.31)
**Sex Partners in last Twelve Months**	
One (Ref)	
Two or more	3.4 (0.6 to 16.4)
**Knowledge of Anything that Could Harm Baby during Pregnancy**	
Yes (Ref)	
No	14.8 (2.32 to 94.9)^*^
**Attitude towards Alcohol Use During Pregnancy**	
Negative (Ref)	
Fair attitude	1.16 (0.03 to 7.17)
Positive attitude	8.4 (0.91 to 107.4)
**My Family would not approve of me drinking while pregnant**	
Disagree (Ref)	
Agree	0.2 (0.04 to 0.93)^*^
**My Friends and Community would approve of me drinking when pregnant**	
Disagree (Ref)	
Agree	0.5 (0.11 to 2.67)
**Ever received Information on drinking during pregnancy**	
Yes (Ref)	
No	2.8 (0.46 to 17.7)
**Knowledge of Dangers of Alcohol Use during Pregnancy**	
No (Ref)	
Number of dangers	0.49 (0.08 to2.93)
Number of observations = 80	
LR chi2 (17) = 27.39	
Prob > chi2 = 0.0374	
Pseudo R2 = 0.3031	

^*^p < 0.05

Variables that predicted binge drinking included knowledge and attitude

## Discussion

In this cross sectional study of 420 women seeking antenatal care in selected health facilities in Gulu, Kitgum and Pader districts in post conflict Northern Uganda, 23.6% reported current maternal alcohol use (any amount), 194 (46.2%) reported lifetime alcohol abstinence and 33 women reportedly abandoned alcohol use on pregnancy recognition.

The relatively high (23.6%) proportion of maternal alcohol use reported in this study could be attributed to availability of cheap alcoholic beverages that are served on various functions such as market days and clan gatherings. Being a post conflict environment, this region also experienced scale up of alcohol promotions as new markets opened up and both local and international alcohol companies sought to expanded their clientele [7, 37].

The prevalence of alcohol use during the current pregnancy in this study is higher than the proportion (16%) reported in western Uganda [38] and almost the same as the proportions (25%) reported among urban women in Kampala [27]. This proportion is also much lower than the 59.28% reported by pregnant Nigerian women [19]. It is higher than the reported 10 and 15% of pregnant women in Canada and the United States [39].

Binge drinking and frequent drinking were less commonly reported by pregnant mothers in this study. This is disimilar to other studies conducted in conflicted affected regions where Alcohol Use Disorder has been reportedly common [37]. Binge drinking is occasionally done during festivities and thus not as common. This is not surprising as related studies reported binge drinking during pregnancy to range from 0.2 to 13.9% [40]. In a recent study in Zambia, pregnant women at risk of problem drinking were 21.2% [35].

Knowledge and attitude were important predictors of alcohol use during pregnancy. Women who agreed to the question my Family would not approve of me drinking while pregnant were less likely to drink while those who agreed to the statement that My friends and Community would approve of me drinking when pregnant were more likely to drink. This relates to study in a tribal dominated district among pregnant Indian women who reportedly were unable to stop drinking during pregnancy because it was generally accepted by their communities [36]. Interventions seeking to reduce alcohol use during pregnancy should target not only pregnant women but generally women in the reproductive age group and their influencers such as family members and significant others.

Other post conflict related studies have also reported alcohol use as form of self- medication or coping strategy. This is aggravated by poor living conditions and challenges in accessing healthcare [41]. Healthcare workers should make deliberate efforts to identify women abusing alcohol due to trauma and provide them with counseling.

There seems to be a paradoxical relationship between specific knowledge and maternal alcohol use in this study. The more dangers of alcohol use during pregnancy a respondent cited the more they were likely to drink. Perhaps some for these women had reached addiction stage and are unable to control their drinking or that alcohol being a social drink they could be receiving information on alcohol use from their social networks such as drinking places. Also, these women may not have been victims of the dangers of drinking during pregnancy. These results are similar to what was found among Canadian women for whom specific knowledge including knowledge that drinking during pregnancy can lead to lifelong disabilities in a child predicted maternal drinking [24]. But also, adequate knowledge (mentioned 4 or more effects of alcohol on mother and baby) was reported by very few (*N* = 10, 2.4%). The high levels of ignorance of repercussions of drinking during pregnancy might be responsible for high levels of alcohol use during pregnancy.

Health workers were the most common source of information on dangers of drinking during pregnancy (*N* = 178, 75.1%) followed by friends (*N* = 65, 27.4%). This is higher than 33.5% of pregnant women in Ghana who got information on maternal drinking in ANC clinics [20] and 62.29% women told by a health professional in Nigeria [19]. Whereas this statistic is impressive, it falls short of expectation that all pregnant women seeking care at health facilities should be educated about all possible risks of harm to pregnancy including alcohol use.

Strengths of our study are that it has a large sample size and provides contemporaneous prevalence data on alcohol use during pregnancy in a post conflict setting and investigates predictors of alcohol use (any amount), frequent and heavy drinking. However, this study has some limitations, it was a cross sectional study thus cause and effect could not be established. Also, this study could have been affected by under reporting due to social desirability and recall bias. Most of the alcohol consumed was locally brewed and for some of the homemade brews, the alcohol by volume is unknown so it was hard to convert them into the US standard drink. None the less, literature maintains that there is no safe amount of alcohol consumption during pregnancy. Some confidence intervals were very wide perhaps because some responses had few observations such as women who never consumed alcohol before pregnancy but started consuming after pregnancy recognition. We recommend future studies to explore this further.

## Conclusion and recommendations

This study indicated that alcohol use (any mount) during pregnancy is high while alcohol dependence, problematic and hazardous drinking is low. Knowledge and attitude were important predictors of alcohol use. While alleviating alcohol use, development partners and relevant government departments should consider interventions that increase knowledge and risk perception on maternal drinking. Other risk factors that predict maternal drinking such as prior alcohol use, residence and parity should be mitigated or eliminated. Interventions seeking to reduce alcohol use during pregnancy should target not only pregnant women but generally women in the reproductive age group and their influencers such as family members and significant others.

Given that this is one of a few studies that have assessed drinking during pregnancy in a post conflict area, the results call for urgent attention to the need for specifically targeted interventions to reduce alcohol use among women of child bearing age and pregnant women in particular. This will reduce related risks to the mother and the baby.

## Data Availability

All relevant data are within the paper. If further data needed, it could be accessed from the first author upon request via email at agiresaasi@gmail.com.

## Abbreviations

ABV: Alcohol by Volume
ANC: Antenatal Care
AUDIT: Alcohol Use Disorders Identification Test
DHO: District Health Officer
FASD: Fetal Alcohol Spectrum Disorders
LBW: Low Birth Weight
LRA: Lord’s Resistance Army
ODK: Open Data Kit
OR: Odds Ratio
PTB: Preterm Births
SPSS: Statistical Package for Social Sciences
UNCST: Uganda National Council for Science and Technology
WHO: World Health Organization
